# RExPrimer: an integrated primer designing tool increases PCR effectiveness by avoiding 3' SNP-in-primer and mis-priming from structural variation

**DOI:** 10.1186/1471-2164-10-S3-S4

**Published:** 2009-12-03

**Authors:** Jittima Piriyapongsa, Chumpol Ngamphiw, Anunchai Assawamakin, Pongsakorn Wangkumhang, Payiarat Suwannasri, Uttapong Ruangrit, Gallissara Agavatpanitch, Sissades Tongsima

**Affiliations:** 1 Genome Institute, National Center for Genetic Engineering and Biotechnology, Pathumthani, Thailand; 2 Division of Molecular Genetics, Department of Research and Development, Faculty of Medicine, Siriraj Hospital, Bangkok, Thailand; 3 Department of Pharmacology, Faculty of Pharmaceutical Sciences, Chulalongkorn University, Bangkok, Thailand

## Abstract

**Background:**

Polymerase chain reaction (PCR) is very useful in many areas of molecular biology research. It is commonly observed that PCR success is critically dependent on design of an effective primer pair. Current tools for primer design do not adequately address the problem of PCR failure due to mis-priming on target-related sequences and structural variations in the genome.

**Methods:**

We have developed an integrated graphical web-based application for primer design, called RExPrimer, which was written in Python language. The software uses Primer3 as the primer designing core algorithm. Locally stored sequence information and genomic variant information were hosted on MySQLv5.0 and were incorporated into RExPrimer.

**Results:**

RExPrimer provides many functionalities for improved PCR primer design. Several databases, namely annotated human SNP databases, insertion/deletion (indel) polymorphisms database, pseudogene database, and structural genomic variation databases were integrated into RExPrimer, enabling an effective without-leaving-the-website validation of the resulting primers. By incorporating these databases, the primers reported by RExPrimer avoid mis-priming to related sequences (e.g. pseudogene, segmental duplication) as well as possible PCR failure because of structural polymorphisms (SNP, indel, and copy number variation (CNV)). To prevent mismatching caused by unexpected SNPs in the designed primers, in particular the 3' end (SNP-in-Primer), several SNP databases covering the broad range of population-specific SNP information are utilized to report SNPs present in the primer sequences. Population-specific SNP information also helps customize primer design for a specific population. Furthermore, RExPrimer offers a graphical user-friendly interface through the use of scalable vector graphic image that intuitively presents resulting primers along with the corresponding gene structure. In this study, we demonstrated the program effectiveness in successfully generating primers for strong homologous sequences.

**Conclusion:**

The improvements for primer design incorporated into RExPrimer were demonstrated to be effective in designing primers for challenging PCR experiments. Integration of SNP and structural variation databases allows for robust primer design for a variety of PCR applications, irrespective of the sequence complexity in the region of interest. This software is freely available at http://www4a.biotec.or.th/rexprimer.

## Background

Polymerase Chain Reaction (PCR) is a common laboratory technique in biological and medical sciences, with a wide range of applications such as DNA cloning, DNA resequencing for single nucleotide polymorphism (SNP) discovery and quantification of gene expression. In the design of any PCR experiment, the first step of designing oligonucleotide primer pairs is crucial for the success of the experiment. Selection of inappropriate primers can result in no amplification (PCR failure) or amplification of non-targeted regions (mis-priming). Therefore, the primer pair is tailored to be specific to the desired target sequence.

The design of target-specific primers for PCR experiments typically requires consideration of different types of genomic information besides the target DNA sequence, such as repetitive DNA elements, intron/exon boundaries, and SNPs, which must be retrieved from various databases. All information is then combined to construct a sequence template for a primer design program. To confirm their specificity, designed primers are usually aligned against the corresponding genome sequence using tools like BLAST [1], BLAT [2], and PrimerBLAST in NCBI. If the aligned results return multiple hits, then the primers are regarded as non-specific and have to be redesigned by constructing a new template avoiding previously considered primer-binding regions. The whole process needs to be repeated manually until the desired primers are found. Thus, manually assigning an appropriate primer pair can be a tedious and time-consuming process, especially when high-throughput assays are required.

To resolve this situation, a number of automated primer designing tools have been developed based on Primer3 [3] as web applications. These programs include SNPbox [4], ELXR [5], ExPrimer [6], MutScreener [7], EasyExonPrimer [8], PrimerZ [9], and others. Most existing tools are limited to specific regions of the human genome and hence they are not flexible enough for users to choose desired target genomic regions (e.g. promoter, intron/exon, SNP) to be amplified. After primers have been picked, most of the tools use UCSC In-Silico PCR [10] to verify the uniqueness of desired primer pair; however, when the selected primers perform poorly, the information from these unsuccessful primer pairs is not considered by these tools for redesigning primers.

While these programs provide some solutions related to the aforementioned primer design process, they are not always able to effectively design primers for two main problems, namely 1) no amplification due to severe mismatching, or lack of target and 2) mis-priming (non-specific binding besides the target sequence). These two problems lead to increased PCR failure rate [11]. The first problem may arise because of unexpected SNPs in the primer 3' end (SNP-in-Primer). Alternatively, insertion/deletion (indel) polymorphisms may exist which either alter the length of the desired target, or prevent primer binding to the desired target. In some cases, the target sequence may be entirely absent, e.g. copy number variation (CNV) covering large stretches of DNA. Three prominent primer-designing tools that attempt to avoid SNP-in-Primer include ExonPrimer [12], EasyExonPrimer [8] and VariantSEQr [13]. However, it is becoming increasingly clear that CNVs are also common and population-specific [14]. Length and copy number polymorphisms can also cause both mismatching and non-primer binding to the desired target, yet these genetic variants are not considered by current primer designing tools. The second problem of mis-priming arises from the structural complexity of the genome. The human genome has many layers of repetition ranging from widespread chromosome segmental duplications, to gene families and pseudogenes to numerous repetitive elements (e.g. SINES, LINES, satellite sequences, etc.), which can all contribute to mis-priming [15]. To our knowledge, there are no primer designing tools that can simultaneously address both of these issues.

To address these issues, we present a graphical web-based tool, named RExPrimer, which allows users to automatically design PCR primer pairs for amplifying human genomic sequence without leaving the website. RExPrimer uses Primer3 as the design core, since this open source software has been continuously adopted by research communities as the *de facto *standard [4-9]. The novel modules that address the aforementioned problems were created on top of the Primer3 core by locally incorporating annotated human genomic sequences. RExPrimer assesses primer candidates for SNP-in-Primer, indel polymorphisms, CNV, and related target sequences (e.g. pseudogenes) by crosschecking with local databases. Large integrated SNP and indel polymorphism databases can notify SNP-in-Primer effects, while information from structural variation databases identifies possible mis-priming. RExPrimer uniquely offers a redesign module for assisting users to correct the notified problems.

## Results

### Input processing

RExPrimer offers three modules of primer design: 1) for resequencing genomic DNA (promoter, exon/intron boundary, any genomic region), 2) for SNP genotyping (gene based and region based), and 3) for oligonucleotide checking. The following types of identifiers are supported as input: HUGO gene name, NCBI Gene ID, and chromosomal locations. Since there are also other interesting non-gene regions, such as regulatory regions and intergenic regions, our program also supports arbitrary genomic regions as input based on chromosome location. For the SNP genotyping module, the program allows one additional input format as a SNP ID (rs-number) or set of SNP IDs. Because this application supports batch design for SNP genotyping primers, it can enhance productivity of high-throughput SNP sequencing projects.

After the input is received, the local human genome database module is interrogated and the corresponding target sequence information, including the genomic sequence, annotations (e.g., promoters, introns, exons) and polymorphisms is subsequently identified and retrieved using query language. When a gene name is used as an input, more than one associated sequence, such as splicing variants may be found in the database. In this case, users have an option to choose the desired isoform. Then, the program supplies the option of selecting the gene regions to be amplified: whole gene (only exons or all gene regions), region of interest (specified by intron/exon number e.g. from intron/exon x to intron/exon y, list of non-contiguous intron/exon).

**Figure 1:**
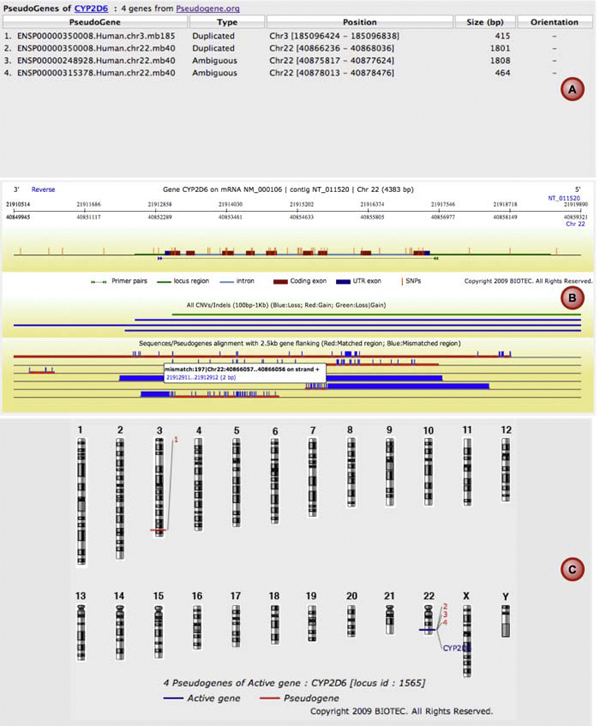
**Detection of *CYP2D6 *pseudogenes**. List of *CYP2D6 *pseudogenes (A) is present along with the multiple alignment of these pseudogene sequences and *CYP2D6 *(B) as well as chromosomal view of pseudogene locations (C).

Non-unique PCR primers are one of the main factors that lead to PCR failure. Multiple regions of homologous, or near-homologous sequence in the genome can compete with the target sequence for primer hybridization leading to PCR artifacts. The human genome contains many pseudogenes, which can have near-perfect homology to their functional counterparts. To avoid designing non-specific primers for genes with pseudogene counterparts, the input sequence's pre-defined pseudogene sequences are retrieved from the pseudogene database module. Multiple sequence alignment of these pseudogene sequences and target gene including alignment scores by the MUMmer program [16] are displayed (Figure 1). The target sequences where primer pairs could not be successfully designed owing to potential mis-priming are highlighted. Users can take this information into consideration when selecting the gene regions to be amplified. Optionally, RExPrimer can automatically exclude ambiguous target regions based on similarity with pseudogene copies. In the case that whole target genes are highly similar to their pseudogenes and no unique region could be identified, users could analyze the gene structure and look for unique sequences flanking the sequence of interest. For DNA resequencing, it is suggested that a primary primer pair which can discriminate the target gene from the corresponding pseudogene is designed. Then, nested primers for sequencing can be generated which do not have to be discriminatory. In this paper, a case study of the *CYP2D6*, which has highly similar pseudogenes is illustrated.

After all of the target DNA sequence information is retrieved from the local database module, it is passed as input to Primer3 for the automatic generation of primer pairs. The RExPrimer user interface allows the users to specify parameters for Primer3 primer selection, such as product size, melting temperature (Tm), GC content. If desired, each designed primer could be screened against a repeat database to reduce nonspecific priming by choosing this available option. The amplicon size is user-defined according to the constraints of the experiment, e.g. accurate sequencing limits amplicons to a few hundred base pairs. If the input sequence is larger than a user-specified product size limit, the program automatically subdivides the template sequence into smaller segments with user-defined segment overlap size in order to ensure that the overlapping sequences can give high quality sequencing data. Primers are then designed separately for each overlapping fragment.

**Figure 2:**
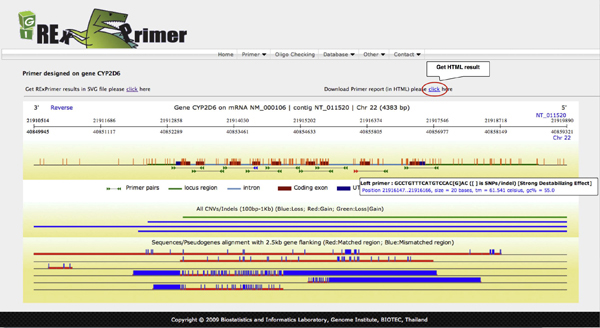
**The SVG graphical output displaying resulting primer pairs with other genomic features**. The designed primer pairs are represented by double-headed arrows along with gene structure (intron/exon) and other genomic features (pseudogenes, indels, CNVs, etc.). Pseudogenes and CNVs/indels are shown as multiple sequence alignments with the target gene. SNP-in-Primers are demonstrated with different colors according to the degree of destabilizing effects introduced by such SNPs. Green color presents primer without SNPs inside. Blue color represents primer with SNP in any position but not within 7 bp of the 3'end while red color specifies primer with SNP found within 7 bp of the 3'end. Each designed primer pair can be linked-out to a redesign module.

Sequence variation in the 3' end, particularly the last three bases can severely disrupt primer hybridization and thus decrease PCR efficiency to the point of PCR amplification failure [17]. Hence, primers should be designed to avoid regions of known polymorphisms based on the information in available SNP databases. To avoid SNP-in-Primer and to design primers for SNP genotyping, RExPrimer utilizes a SNP database module. This SNP database module enables effective primer design for SNP genotyping and SNP identification. For resequencing primers, designed primers are checked for SNP-in-Primer regions, and any SNPs present are notified through a color-coded graphical display output module, the color reflecting the level of the mismatching primer-destabilizing effects [18] (Figure 2). With this information, users can adjust primer positions to avoid SNP-in-Primer. In addition, information from different SNP databases provides clues for population-specific primer design, i.e., SNPs found only in some populations will give SNP-in-Primer effects for those populations, and not in others.

### Output report

**Figure 3:**
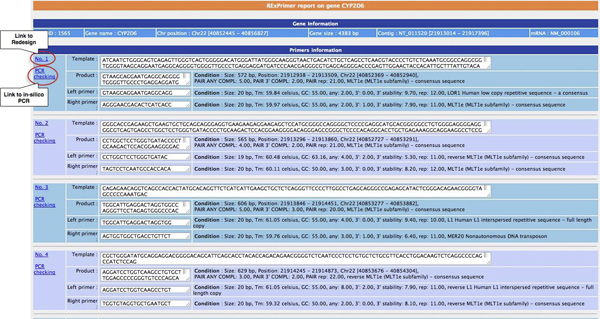
**HTML report of designed primers**. The designed primer sequences are shown with assigned PCR conditions. Redesign module and uniqueness test by In-Silico PCR are provided as the links from this report.

Results reported from RExPrimer include two parts: SVG graphical display and HTML primer information summary. The graphical representation displays resulting primer pairs with their respective locations in the target gene in the same window (Figure 2). If present, SNP-in-Primers, pseudogenes, CNVs, and indel polymorphisms are also displayed. In addition to the visual summary, the designed primer text summary describing Primer3 parameters and primer conditions is also generated in HTML format (Figure 3). Each designed primer pair can quickly be verified for uniqueness against the entire genome using the locally installed UCSC In-Silico PCR in RExPrimer.

When strong primer-destabilizing effects such as SNP-in-Primer are reported, users can immediately redesign primers without repeating the same procedure again. Optionally, the users can directly specify the approximate range of primer or target region, e.g., to avoid regions of high SNP density. The redesign of primer pairs can then be forced to a confined user-specified region.

### Case study: CYP2D6

To validate whether RExPrimer is effective in designing primers in challenging PCR applications, RExPrimer was used to design primers for the Cytochrome P450 2D6 locus (*CYP2D6*), which is thought as one of the most important enzymes for the metabolism of many clinically used drugs. *CYP2D6 *is highly polymorphic, which can be expressed as variation in *CYP2D6*-related drug metabolism among individuals. Besides a growing number of SNPs, numerous CNVs have been reported worldwide for this locus. Therefore, extensive population-wide study of genetic variations at *CYP2D6 *has medical importance. There are several problems that could interfere with the accuracy of genotyping, including:

• Pseudogenes (*CYP2D7P *and *CYP2D8P *which contain almost 98% of sequence homology to *CYP2D6 *[19])

• CNVs from unequal crossing-over

• SNPs reported to influence the enzyme activity

Existing primer design tools could have failed to consider the effect from *CYP2D6 *pseudogenes, SNPs, and CNVs. Mis-priming to pseudogenes can lead to artifactual PCR. SNPs and CNVs can lead to mismatching and non-primer binding to the desired region. The number of potential primers that are specific to *CYP2D6 *are thus limited.

**Figure 4:**
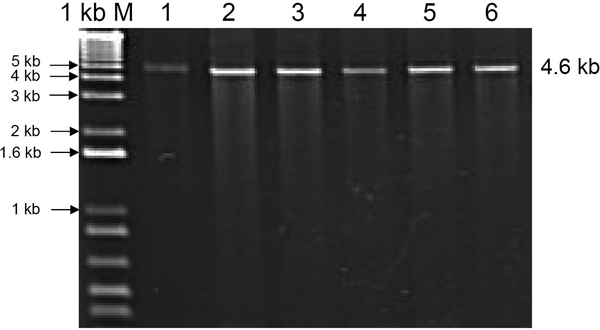
**PCR amplification of *CYP2D6***. Agarose gel electrophoresis of *CYP2D6 *gene sequences amplified by PCR. Lanes 1-6 show amplification of the *CYP2D6 *whole gene using primers CYP2D6_1F and CYP2D6_1R (shown in Table 1) from different human samples. 1 kb M is DNA ladder marker.

**Figure 5:**
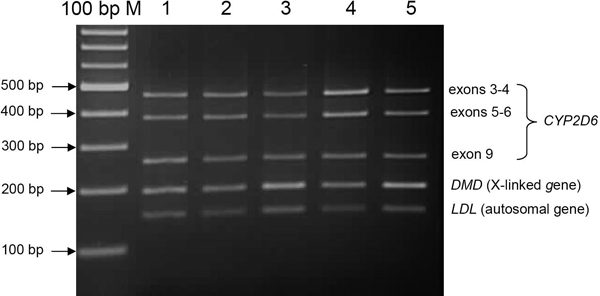
**Semiquantitative analysis of *CYP2D6 *copy number variations**. Successful penta-plex PCR products of *CYP2D6 *analysis were shown. Lanes 1-5 show amplification of the *CYP2D6 *exon sequences, *LDL*, and *DMD *from different human samples using five primer pairs listed in Table 1. *LDL *is an internal standard for common two copies (autosomal gene) and *DMD *is internal standard for one copy in men (X-linked). 100 bp M is DNA marker. *DMD *= dystrophin. *LDL *= low density lipoprotein.

In this study, primers were cautiously designed by considering every possible problem mentioned above using RExPrimer. The primers designed by RExPrimer for the *CYP2D6 *locus are shown in Table 1. PCR experiments were then carried out to verify the effectiveness of the designed primers. RExPrimer detected four pseudogenes which showed high similarity with *CYP2D6 *before the primer designing step (Figure 1). In order to detect every SNP on the active *CYP2D6*, the information in Figure 1 guided us to perform whole gene amplification which can separate the active gene from the pseudogene counterparts (Figure 4). Once the active gene was isolated, the SNP genotyping can be further conducted. For CNVs, we chose semiquantitative analysis [20] to detect the different number of CNVs by quantifying the resulting PCR products. Samples were collected from volunteers in accordance with a local ethical committee in an ongoing study of human variation, details of which will be published elsewhere. Penta-plex PCR was developed from genomic DNA and the resulting products were analyzed with DHPLC (Denaturing High Performance Liquid Chromatography) in semiquantitative mode. Three regions from *CYP2D6 *gene were selectively amplified covering exons 3 to 4, exons 5 to 6, and exon 9. The low density lipoprotein (*LDL*) and dystrophin (*DMD*) genes were used as internal controls (Figure 5). The intensities of the *CYP2D6 *PCR products when compared with the internal standards *DMD *and *LDL *by DHPLC indicate that *CYP2D6 *copies are present in each individual (data not shown). These results demonstrate that the primers designed by RExPrimer are suitable for CNV genotyping.

**Table 1:** The designed primers for *CYP2D6 *analysis obtained from RExPrimer

Name	Sequence 5' → 3'	Purpose
CYP2D6_1F	GGCCTACCCTGGGTAAGGGCCTGGAGCAGGA	whole gene amplification
CYP2D6_1R	CTCAGCCTCAACGTACCCCTGTCTCAAATGCG	

CYP2D6_2F	GAGACTCCTCGGTCTCTCG	penta-plex PCR
CYP2D6_2R	TAATGCCTTCATGGCCACGCG	

CYP2D6_3F	AGGCCTTCCTGGCAGAGATGAAG	penta-plex PCR
CYP2D6_3R	CCCCTGCACTGTTTCCCAGA	

CYP2D6_4F	CCAGCCACCATGGTGTCTTTG	penta-plex PCR
CYP2D6_4R	GCCTCAACGTACCCCTGTCTC	

DMD_F	TTGTCGGTCTCCTGCTGGTCAGTG	one-copy internal standard in penta-plex PCR
DMD_R	CAAAGCCCTCACTCAAACATGAAGC	

LDL_F	TACAAGTGCCAGTGTGAGGAAG	two-copy internal standard in penta-plex PCR
LDL_R	GTGCAAAGTTCAGAGGATGAAACT	

Rows 2-6 represent semiquantitative analysis of CNV

## Discussion

In this study, we have developed a comprehensive tool for PCR primer design which covers a broad range of functionalities including resequencing target genes and SNP genotyping. The primer design pipeline is composed of three key components: 1) pseudogene detection, 2) primer design core, and 3) SNP-in-Primer/genomic variation notification. Additional file 1 presents the feature comparison among existing primer designing software.

RExPrimer utilizes the publicly available sequence information including the human genome and annotation database, SNP databases, and the pseudogene database. By incorporating these databases and the Primer3 program, reliable and accurate primer designs can be achieved. The current pseudogene database module comprises of approximately sixteen thousand pseudogenes [21] while the genomic variation module (indels, CNVs, inversions) consists a total of thirty-eight thousand entries [15]. SNP database hosts more than nineteen million common and population specific SNPs from various populations, which is larger and more comprehensive than existing primer design programs [8,12,13].

Most primer design tools verify the uniqueness of the PCR target sequence by using UCSC In-Silico PCR [10] after the primer candidates are picked. However, if the desired target has no unique segments, the primer specificity search would run indefinitely, thus slowing down the primer generation procedure. RExPrimer avoids this problem by excluding target regions shared with pseudogenes and other related sequences before primers are generated. The program also takes care of other genomic variation issues. This was demonstrated in the *CYP2D6 *case study provided in the result section.

RExPrimer appends several key features before and after the primer design process using Primer3, enabling the selection of unique primer sequences and reliably amplifiable targets, which other currently available software cannot match. However, with the caveat that the primers designed are unique and the targets are amplifiable (no CNV or SNP-in-Primer), no extra claims for the actual performance of the primers beyond what is predicted by Primer3 are made. Hence, we have not attempted to determine the success rate of RExPrimer for designing primers, since the cost of performing multiple PCR experiments is not justified. Finally, RExPrimer has a major strength on its graphical web interface, especially the gene structure visualization, which makes RExPrimer intuitive and user friendly.

### Future development

Currently, RExPrimer can offer the oligonucleotide primer design for human genomic sequence in which SNP and pseudogene data are largely available. In the future, the program and locally built databases will be expanded to support primer generation for different organisms once their pseudogene and polymorphism data are readily available. To enhance the quality of primer design, the local SNP databases will be automatically updated to make use of the latest information, including as wide a range of population diversity as available.

## Conclusion

RExPrimer is a one-stop tool for PCR primer design, which can support high-throughput resequencing and mutation screening research. The incorporation of large SNP and genomic variation databases make it possible to efficiently detect sequence variation in the designed primers that might cause PCR failure. The notification system of target pseudogenes before the primer design step helps users to select the appropriate target regions, which can significantly shorten the design process. RExPrimer is shown in this study to be indeed effective for designing primers for *CYP2D6*. We expect that RExPrimer is able to fill the gap and accomplish current needs for automated primer design procedure.

## Methods

**Figure 6:**
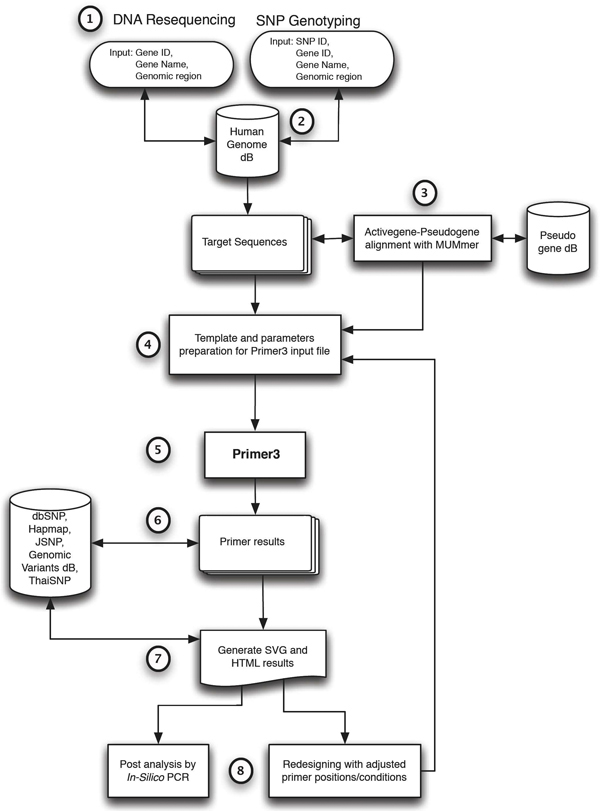
**The organization of RExPrimer**. The workflow of RExPrimer following the steps described in methods section is displayed.

RExPrimer is a web-based application that was written in Python language. The web interface was created by Python Webware framework [22]. This application was deployed on Sun UltraSparc V880 running Solaris 10 operating system located at the National Center for Genetic Engineering and Biotechnology (BIOTEC), Thailand. SVG supported web browser, e.g., Firefox, Safari, iPhone2.2.1, is required. IE is not supported due to JAVA script incompatibility. The overall architecture of RExPrimer is presented in Figure 6. The following steps describe the architecture.

1. Users provide input parameters required to design primer pairs such as gene name, SNP ID, genomic location.

2. The input parameters are used to query the targeted sequence from the local human genome sequence database, which was retrieved from NCBI and stored locally on our server.

3. The target sequence is checked using MUMmer [16] for pseudogene match from locally constructed pseudogene database which was retrieved from [21].

4. RExPrimer prepares the input file for Primer3 from the primer conditions and target sequence. If the target sequence is greater than the target size parameter set in Primer3, the system will construct a set of overlapping fragments that covers the entire target sequence.

5. Primer3 processes the input files prepared in step 4 and generates the primer pair results in Primer3 format.

6. The primer pair results are then crosschecked against different locally constructed SNP and genomic variation databases covering a wide range of human SNP variation, CNV, and indel polymorphisms.

7. Primer pairs are visualized on the locus-specific region using scalable vector graphics (SVG). The software also provides primer information in HTML format (see Figure 3) as a link on the resulting graphic page (see Figure 2).

8. Each resulting primer pair can be validated for uniqueness using local In-Silico PCR (see Figure 3). All of the designed primer pair results presented on the graphic and HTML page can be linked-out to a RExPrimer redesign module (see Figure 2, 3).

RExPrimer stores public sequence information and genomic variant information in order to accelerate the speed of processing and allow the user to seamlessly unify the required information used in RExPrimer. These databases are hosted on MySQLv5.0, which offers several important services to RExPrimer. The human genome sequence database module was downloaded from NCBI build 36.3 to be used as template sequence as well as providing gene organization information. The SNP database module comprises of common and population-specific SNPs from various databases, namely NCBI dbSNP [23] build 129, HapMap [24] public release 27, JSNP [25] release 35, and ThaiSNP [26] release 2, which can notify SNP-in-Primers. The genomic variation module for human genome build 36 (hg18) consists of indels, inversions, CNVs data obtained from Database of Genomic Variant [15]. Furthermore, the pseudogene database [21] for human genome build 36 was incorporated into the system to assist in detecting potential mis-priming.

## Competing interests

The authors declare that they have no competing interests.

## Authors' contributions

JP wrote the manuscript and designed RExPrimer. CN developed RExPrimer and wrote the manuscript. AA wrote the manuscript, designed RExPrimer and *CYP2D6 *experiments. PW developed SNP-in-Primer module and wrote the manuscript. PS and GA designed and performed *CYP2D6 *experiments. UR designed the web interface of RExPrimer. ST wrote the manuscript, designed and oversaw the development of RExPrimer. All authors read and approved the final manuscript.

## Note

Other papers from the meeting have been published as part of *BMC Bioinformatics* Volume 10 Supplement 15, 2009: Eighth International Conference on Bioinformatics (InCoB2009): Bioinformatics, available online at http://www.biomedcentral.com/1471-2105/10?issue=S15.

## Supplementary Material

Additional file 1 **The feature comparison of existing primer designing software**. The table displays the features provided in currently available primer designing software compared with those present in RExPrimer program. The cells with bold italic letters indicate unique features offered by RExPrimer. Click here for file
