# Supplementary material for: RExPrimer: an integrated primer designing tool increases PCR effectiveness by avoiding 3' SNP-in-primer and mis-priming from structural variation
Source: BMC Genomics. 2009 Dec 3;10(Suppl 3):S4. doi: 10.1186/1471-2164-10-S3-S4 (PMC2788391; doi:10.1186/1471-2164-10-S3-S4)
Supplement: Additional file 1 — The feature comparison of existing primer designing software. The table displays the features provided in currently available primer designing software compared with those present in RExPrimer program. The cells with bold italic letters indicate unique features offered by RExPrimer. [file 1471-2164-10-S3-S4-S1.pdf]

Supplement Table 1 - The feature comparison of existing primer designing software

Feature		RExPrimer	PrimerZ	MutScreener	EasyExonPrimer	VariantSEQr	ELXR	SNPbox	ExonPrimer	Genomic Primer
**Software Functionalities**	**sequence information retrievals and annotations**	***GenBank (Local)***	Ensembl and GenBank (Remote)	GenBank (Remote)	UCSC Genome Browser (Remote)	N/A	NCBI RefSeq (Remote)	GenBank (Remote)	N/A	N/A
	**promoter resequencing**	Available	Available	Available	N/A	Available	N/A	Available	N/A	N/A
	**continuous genomic DNA resequencing**	Available	N/A	Available	N/A	N/A	N/A	Available	N/A	N/A
	**combining two short exons as one template**	Available	N/A	Warning	N/A	N/A	N/A	N/A	Available	N/A
	**overlapping primers for large exons**	Available	Available	Available	Available	Available	Available	N/A	Available	N/A
	**redesign**	***Allowing users to avoid the notified problems for the next design***	Primer3 parameters can be adjusted to improve the designed primers	N/A	N/A	N/A	N/A	N/A	N/A	N/A

Feature		RExPrimer	PrimerZ	MutScreener	EasyExonPrimer	VariantSEQr	ELXR	SNPbox	ExonPrimer	Genomic Primer
**Primer Specificity**	**avoiding non-target homologous sequences e.g. pseudogenes**	***Avoiding by using information from Pseudogene database***	N/A	N/A	N/A	N/A	Masking using RepeatMasker	Masking using RepeatMasker	Masking using BLAT	N/A
	**SNPs/Indels allocated in primers**	***Warning/notifying destabilizing effect for redesign decision***	N/A	N/A	Avoid	Avoid	N/A	N/A	Avoid	N/A
	**CNV/Indels allocated in primers**	***Warning***	N/A	N/A	N/A	N/A	N/A	N/A	N/A	N/A
	**Specificity checking**	In-Silico PCR	In-Silico PCR, NCBI BLAST	N/A	In-Silico PCR	Experimental testing listed in NCBI ProbeDB	N/A	N/A	N/A	N/A
**Appearance**	**Input format**	gene name, gene ID, genomic location	gene name, Ensembl ID	GenBank accession, gene ID, input sequence	RefSeq ID, gene name, keyword	gene ID, genomic location, keyword	RefSeq ID, input sequence	GenBank accession, input sequence	input sequence	input sequence, gene name
	**Visualization for user guidance**	***Graphical view of gene structure, genome annotation (SNP, CNV, pseudogene, active gene-pseudogene alignment, etc.) and resulting primers***	Graphical view of gene structure and genome annotation	N/A	N/A	N/A	Graphical view of predicted gene structure obtained from sequence alignment	N/A	N/A	N/A

**Feature**		**RExPrimer**	**PrimerZ**	**MutScreener**	**EasyExonPrimer**	**VariantSEQr**	**ELXR**	**SNPbox**	**ExonPrimer**	**Genomic Primer**
	**Output format**	SVG graphic viewer and HTML	Text format	Text format and tabular format	Text format	Text format and graphic viewer	Text format and graphic viewer	Text format and graphic viewer	Text format	Text format
